# The approved pediatric drug suramin identified as a clinical candidate for the treatment of EV71 infection—suramin inhibits EV71 infection *in vitro* and *in vivo*

**DOI:** 10.1038/emi.2014.60

**Published:** 2014-09-03

**Authors:** Peijun Ren, Gang Zou, Benjamin Bailly, Shanshan Xu, Mei Zeng, Xinsheng Chen, Liang Shen, Ying Zhang, Patrice Guillon, Fernando Arenzana-Seisdedos, Philippe Buchy, Jian Li, Mark von Itzstein, Qihan Li, Ralf Altmeyer

**Affiliations:** 1 Unit of Anti-infective Research, Institut Pasteur of Shanghai, Chinese Academy of Sciences, Shanghai 200031, China; 2 Université Paris Diderot, Sorbonne Paris Cité, Cellule Pasteur, 75013, Paris, France; 3 Institut National de la Santé et de la Recherche Médicale, U1108 & Viral Pathogenesis Unit, Department of Virology, Institut Pasteur, 75724 Paris, France; 4 Institute for Glycomics, Gold Coast Campus, Griffith University, 4222 Queensland, Australia; 5 Department of Infectious Diseases, Children's Hospital of Fudan University, Shanghai 201102, China; 6 State Key Laboratory of Lead Compound Research, WuXi AppTec, Co., Ltd, Shanghai 200131, China; 7 Institute of Medical Biology, Chinese Academy of Medicine Science, Kunming 650118, Yunnan Province, China; 8 Virology Unit, Institut Pasteur in Cambodia, 12201 Phnom Penh, Cambodia

**Keywords:** anti-viral, drug discovery, enterovirus 71, hand, foot and mouth disease, suramin

## Abstract

Enterovirus 71 (EV71) causes severe central nervous system infections, leading to cardiopulmonary complications and death in young children. There is an urgent unmet medical need for new pharmaceutical agents to control EV71 infections. Using a multidisciplinary approach, we found that the approved pediatric antiparasitic drug suramin blocked EV71 infectivity by a novel mechanism of action that involves binding of the naphtalentrisulonic acid group of suramin to the viral capsid. Moreover, we demonstrate that when suramin is used *in vivo* at doses equivalent to or lower than the highest dose already used in humans, it significantly decreased mortality in mice challenged with a lethal dose of EV71 and peak viral load in adult rhesus monkeys. Thus, suramin inhibits EV71 infection by neutralizing virus particles prior to cell attachment. Consequently, these findings identify suramin as a clinical candidate for further development as a therapeutic or prophylactic treatment for severe EV71 infection.

## INTRODUCTION

Hand, foot and mouth disease (HFMD), a contagious infectious disease mostly affecting children under the age of five years, is common in Asia^1,2,3^ and has been particularly prevalent since 2008.^4^ The disease is endemic in other regions but severe forms are rarely observed.^5^ In China alone, over 7.6 million children have been diagnosed with HFMD and more than 2400 of these children have died, since 2008.^4,6,7^ In most cases, symptoms are mild, such as fever, sore throat, malaise, rashes on the hand palms, soles of feet, buttocks and herpangina. However, severe disease, including central nervous system infection, brain stem encephalitis, neurogenic pulmonary edema and cardiopulmonary complications, may occur and is frequently fatal.^2^ human enterovirus serotype 71 (EV71) is the main causal agent of HFMD, and particularly of the severe forms of this disease.^1,2^ Children become susceptible to severe EV71 infections after the loss of maternal antibodies and one to two-year-old children are most at risk.^8^

EV71 is a single-stranded positive-sense RNA virus from the *Picornaviridae* family, genus *Enterovirus* (along with Poliovirus and coxsackievirus species A).^1^ It has a non-enveloped capsid consisting of 60 identical subunits, each containing one copy of each of four viral structural proteins (VP1, 2, 3 and 4), and packages a 7.5 kb genome. EV71 can be classified into genogroups A, B and C, recently identified D, E and F^9^ on the basis of its VP1 gene sequence. Group C is prevalent in East Asia and the C4 genotype currently predominates in mainland China, Vietnam, Cambodia and prevalent in Taiwan and Thailand.^1,10^

During infection, EV71 binds to host cells via viral receptors, such as human scavenger receptor class B, member 2^11^ and P-selectin glycoprotein ligand-1.^12^ Binding to scavenger receptor class B, member 2 triggers the uncoating process,^13^ a series of structural changes occurring in the viral capsid leading to the release of the viral genome into the host cell. Like many other viruses, EV71 also uses cell surface heparan sulfate glycosaminoglycan as attachment receptor to initiate target cell entry.^14^ Also recently, Du *et al*.^15^ demonstrated that cell surface vimentin serves as an attachment site that mediated the initial binding and subsequently increased the infectivity of EV71.

There is currently no specific anti-EV71 drug, and guidelines for the treatment of HFMD are therefore limited to supportive care, antipyretic drugs, intravenous non-immune immunoglobulin and possibly, glucocorticoids.^16^ Type 1 interferons and inhibitors of 3C protease, 3D polymerase and entry inhibitors are candidate drugs for the treatment of EV71 infections. However, no proof-of-concept study has yet been established for these treatments in non-human primate models or clinical trials.

A series of structurally related antiviral compounds known as the Winthrop compounds inhibit picornavirus attachment to host cells and virus uncoating, by binding to a hydrophobic pocket of the capsid.^17^ The Winthrop compound pleconaril, attenuates severe symptoms in EV71-infected mice, although differences in potency between viral isolates were described.^18^ Pleconaril and Winthrop compounds served as scaffolds for the design of pyridyl imidazolidinones.^18^ Two of these compounds, BPR0Z-194 and DBPR-103, have potent antiviral activity, preventing the attachment or uncoating of several enteroviruses, including EV71.^18^ Rupintrivir, or AG7088, a picornavirus 3C protease inhibitor, has potent broad-spectrum activity against human rhinovirus and human enterovirus, including EV71, both *in vitro* and *in vivo*.^19,20^ Ribavirin, which can be incorporated by viral RNA-dependent RNA polymerase, is sometimes used to treat HFMD.^21^ Glycosaminoglycans such as heparin, heparan sulfate and their mimetics have been shown to strongly inhibit EV71 attachment to cells^22^ suggesting that interfering with EV71 binding to glycosaminoglycans could be used as a target for the development of an antiviral.

We investigated whether any United States Food and Drug Administration-approved drugs were of potential value for treating EV71 infection. There are several advantages to focusing on approved drugs: (i) experience in clinical use or data from clinical trials, for pharmacokinetics and toxicity in particular, can significantly decrease development time; and (ii) the physiological roles of the targets of most approved drugs are known, facilitating mechanism-of-action studies and providing valuable information about potential drug–drug interactions.

We identified suramin as a clinical candidate molecule directly binding the EV71 capsid, blocking attachment and entry and decreasing viral replication in susceptible animals. Suramin has been in clinical use for decades,^23^ as a prophylactic and therapeutic agent in children.^24^ Attempts have recently been made to develop the use of suramin in a cancer setting^25^ and as an antiviral agent against human immunodeficiency virus^26^ and hepatitis B virus.^27^ We identified suramin as an inhibitor of EV71 entry and provide the first demonstration of the efficacy of a small molecule in a non-human primate model of EV71 infection.

## MATERIALS AND METHODS

### Cell lines and viruses

RD (human rhabdomyosarcoma) cells were purchased from the American Type Culture Collection (ATCC NO CCL-136). The EV71 isolate Fuyang573 (subgenotype C4a, GenBank accession number: HM064456, isolated from a 2008 epidemic sample in Anhui province) was used throughout this study, unless otherwise stated. EV71 isolates SH12-036 (GenBank accession NO KC570452) and SH12-276 (GenBank accession NO KC570453) were isolated from patient samples in Shanghai, in 2012. SEP-4 (2012 Cambodia EV71 isolate, GenBank accession NO KF543271) was provided by the Virology Unit of the Institut Pasteur in Cambodia. Coxsackie virus A16 (strain shzh05-1, GenBank accession NO EU262658) and poliovirus-1 (Sabin, type I oral poliovirus vaccine) were also used to evaluate antiviral potency. We titrated virus stocks on RD cells, by both microtitration tissue culture infective dose 50% (TCID_50_), according to the Kärber formula and plaque assays in 0.7% carboxymethylcellulose.

### Quantitative reverse transcription polymerase chain reaction (qRT-PCR) for EV71 viral load quantification

We extracted RNA with the TIANamp RNA Extraction Kit for Virus Detection (cat. NO DP315-R; Tiangen Biotech Beijing Co., Ltd, Beijing, China), or the TIANamp N96 Virus RNA Kit (cat. NO DP434; Tiangen Biotech Beijing Co., Ltd) in semihigh-throughput operations. We assessed viral genome load with the Quant One Step qRT-PCR (Probe) Kit (cat. NO FP304; Tiangen Biotech Beijing Co., Ltd) on a 7900HT Fast Real-Time PCR system (Applied Biosystems, Foster City, CA, USA). The VP1 gene was detected with forward primer: 5′-GTT CAC CTA CAT GCG CTT TGA-3′, reverse primer: 5′-TGG AGC AAT TGT GGG ACA AC-3′ and probe: 5′-HEX-TCT TGC GTG CAC ACC CAC CG-TAMRA-3′.^28^ The PCR standard curve was obtained by serial dilution of the defined-titer (TCID_50_/mL) virus stock, and the sample cycle threshold (*C*_T_) number was converted into viral load with this standard curve (Supplementary Figure S1), and viral load is expressed as EV71 genome equivalent.

### Cell viability assay

We evaluated cell viability with the CellTiter-Glo Luminescent Cell Viability Assay Kit (cat. NO G7571; Promega, Fitchburg, WI, USA).

### Drug library and compounds

We screened the United States Drug Collection (1040 compounds) and the International Drug Collection (240 compounds) (MicroSource Discovery Systems Inc., Gaylordsville, CT, USA), searching for compounds active against EV71.

Suramin sodium salt (cat. NO S2671), PPADS (pyridoxal phosphate-6-azo (benzene-2,4-disulfonic acid) tetrasodium salt hydrate, cat. NO P178), vinylsulfonic acid sodium salt (cat. NO 278416) and heparin sodium salt (cat. NO H3393) were purchased from Sigma-Aldrich (St. Louis, MO 63103, USA). DIDS (4,4′-diisothiocyanatostilbene-2,2′-disulfonic acid disodium salt, cat. NO sc-203919) was obtained from Santa Cruz Biotechnology Inc (Santa Cruz, CA 95060, USA). iso-PPADS tetrasodium salt (pyridoxalphosphate-6-azophenyl-2′,5′-disulfonic acid tetrasodium salt, cat. NO 0683), NF 023 (8,8′-[carbonylbis(imino-3,1-phenylenecarbonylimino)]bis-1,3,5-naphthalene-trisulphonic acid, hexasodium salt, cat. NO 1240), NF 157 (8,8′-[carbonylbis[imino-3,1-phenylenecarbonylimino(4-fluoro-3,1-phenylene)carbonylimino]]bis-1,3,5-naphthalenetrisulfonic acid hexasodium salt, cat. NO 2450) and NF 449 (4,4′,4″,4″′-[carbonylbis(imino-5,1,3-benzenetriyl-bis(carbonylimino))]tetrakis-1,3-benzenedisulfonic acid, octasodium salt, cat. NO 1391) were obtained from Tocris Bioscience (Bristol, UK). Sucralfate sodium was obtained from MicroSource Discovery Systems Inc. Suramin used in the monkey study was provided free-of-charge by Bayer Healthcare (Elberfeld, Germany).

### Drug screening

We screened the drug library in 96-well plates, by inoculating 5×10^4^ RD cells per well with 10 µM drugs and incubating at 37°C for 1 h. We then infected cells, at a multiplicity of infection of 0.1, in the presence of 10 µM drugs, at 37°C for 1 h. The cells were then incubated, in the presence of 10 µM drug, at 37°C for 46–48 h, under an atmosphere containing 5% CO_2_. We collected the culture supernatant, extracted the viral RNA and determined viral load by qRT-PCR.

### Antiviral potency assay

The cells and the virus were incubated separately with the compound for 1 h at 37°C. The cells were then infected in the presence of the compound for 1 h and incubated with the compound for 46–48 h. We then evaluated viral load by qRT-PCR. Antiviral potency was also evaluated by microtitration (results expressed as TCID_50_) with a series of concentrations of the compound assessed. Alternatively, plaque assays were carried out, in which we incubated 90% confluent RD cells and virus separately with the compound and then infected cells with 50 plaque forming units EV71 in the presence of the compound.

### *In vivo* anti-EV71 efficacy

The anti-EV71 efficacy of suramin *in vivo* was assessed in 10-day-old Institute of Cancer Research mice^29^ and adult rhesus monkeys,^30^ as previously described. We injected 1×10^7^ TCID_50_ (lethal dose) of the mouse-adapted EV71 strain MP10 (GenBank accession NO HQ712020, genotype C4) intraperitoneally into mice. We then injected 20 or 50 mg/kg suramin dissolved in saline, or saline alone as a placebo, intraperitoneally into the mice, twice daily for seven days. For monkey studies, we intravenously injected 1×10^6.5^ cell culture infective dose 50% (CCID_50_) EV71 FY-23 (GeneBank accession NO EU812515, genotype C4) into the monkey. We then injected 50 mg/kg suramin in saline, or saline alone as a placebo, into the monkeys intravenously on the day before virus challenge and on days 1, 3 and 5 post-challenge. We then assessed serum viral load by qRT-PCR, assessed the neutralizing antibody titer on RD cell as described before,^31^ in neutralizing assay, serum was diluted for eight times.

### Saturation transfer difference nuclear magnetic resonance spectroscopy (STD NMR)

We prepared viral particles for the STD NMR assay by inactivating the virus stock by incubation with 1:2000 (v/v) β-propiolactone (H0168; TCI, Shanghai, China) overnight at 4°C. We then concentrated the viral particles by centrifugation on a 20% sucrose cushion in a Beckman SW28 rotor, at 25 000 r.p.m., 4°C, for 4 h. The pellet was resuspended in phosphate-buffered saline, ultracentrifuged on 10%∼50% sucrose gradients in a Beckman SW41 rotor at 156 000*g* for 16 h at 4°C. The 50% sucrose layer was subjected to centrifugation on a 20% sucrose cushion, and the pellet was resuspended in phosphate-buffered saline.

All NMR experiments were performed on a Bruker 600 MHz Avance spectrometer at 280 K using a conventional ^1^H/^13^C/^15^N gradient cryoprobe system under similar conditions to that previously described.^32^ Deuterium oxide (99.9% deuterium) was purchased from Novachem Pty Ltd (Collingwood, Australia). NMR samples were prepared by mixing EV71 particles and suramin, at a molar ratio of 1:100, in NMR buffer (10 mM NaCl in 20 mM phosphate buffer, pH 7.1).

### Cytochrome P450 (CYP) inhibition assay

CYP inhibition was determined with a marker substrate cocktail. For each reaction, enzyme activities in the presence and absence of the test compound (10, 30 and 100 µM) were measured in duplicate. Known inhibitors for each isoform (*O*-deethylation (CYP1A2), 4′-hydroxylation (CYP2C9), 4′-hydroxylation (CYP2C19), *O*-demethylation (CYP2D6) and 1′-hydroxylation (CYP3A4)), were tested at 3 µM as positive controls.

Incubation mixture containing human microsomes, substrate cocktail and standard inhibitor or test compound was pre-incubated at 37°C for 5 min. The reaction was initiated by adding nicotinamide adenine dinucleotide phosphate. The mixture was incubated at 37°C for 10 min, and ice-cold acetonitrile was added to terminate the reaction. We assessed metabolite generation from the substrate reactions by liquid chromatography-tandem mass spectrometry and peak area ratios for analyte/internal standard. The extent of inhibition was expressed as a % of control activity.

### Cynomolgus monkey plasma pharmacokinetics

We studied the plasma pharmacokinetics of suramin in male cynomolgus monkeys. Three monkeys were given 4.37 mg/kg body weight suramin by intravenous bolus administration, with serial blood sample collection for up to seven days. Plasma samples were obtained by centrifugation (3000*g* for 10 min at 2–8 °C). A liquid chromatography-tandem mass spectrometry method was developed for the quantification of suramin in monkey plasma. Changes in plasma concentration over time were analyzed with a non-compartmental model in WinNonlin software (version 5.2.1; Pharsight, Mountain View, CA, USA), with calculation of the following pharmacokinetic parameters: AUC_0-last_, AUC_0-inf_ (AUC: area under the concentration time curve; AUC_0-last_: AUC up to the last measurable concentration; AUC_0-inf_: AUC curve to infinite time), half-life (T_½_), maximum concentration observed (C_max_), clearance (CL), volumes of distribution calculated either by the steady-state method (Vd_ss_).

### Statistical analysis

In the *in vivo* efficacy test of suramin in monkey, comparisons between the viral load in drug treated group and control groups were performed by the two-way analysis of variance test. A difference with a *P* value of less than 0.05 was considered to be significant.

## RESULTS

### Approved drug library screening

**Figure 1:**
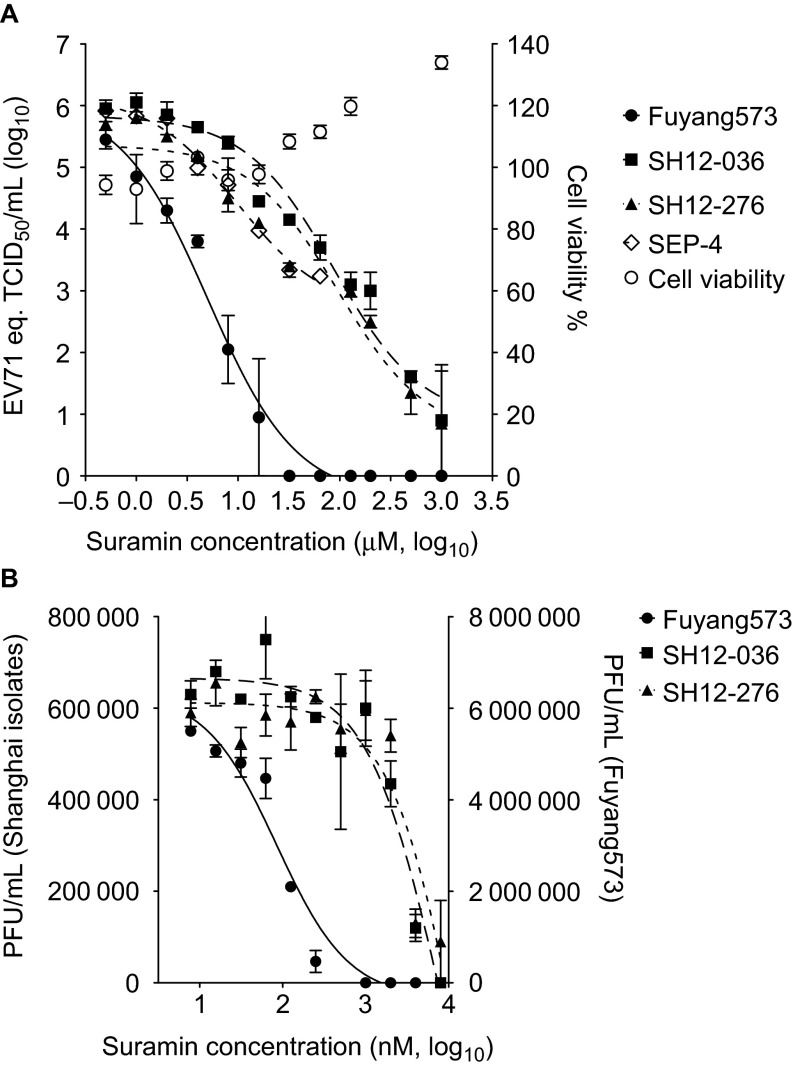
Suramin was identified as an EV71 inhibitor. (**A**) Suramin inhibits the replication of EV71 isolates Fuyang573, SH12-036, SH12-276 and SEP-4, without cytotoxicity. Viral load was measured by quantitative RT-PCR, and expressed as the EV71 genome equivalent to TCID_50_/mL. Data represents the mean±s.e.m. of results of duplicated experiment. (**B**) Suramin reduces the progeny virus yield. Data represents the mean±s.e.m. of results of two independent experiments which are duplicated.

One thousand two hundred and eigthy drugs from the United States and International Drug Collection were screened, at 10 µM, using EV71 genome equivalent reduction in the supernatant of infected RD cells by >1 log_10_ and cytotoxicity less than <25% as readout. Suramin was selected for further analysis based on its inhibition profile and its approval status as a pediatric drug. Suramin inhibited several C4-genotype EV71 isolates (Figure 1A) with a concentration causing 90% inhibition (IC_90_) of 0.93, 3.92, 22.19 and 25.84 µM for the Fuyang573 (Anhui 2008), SH12-036, SH12-276 (Shanghai 2012) and SEP-4 (Cambodia 2012) isolates, respectively. These results were confirmed in an EV71 plaque reduction assay, in which the IC_90_ of suramin was 0.49, 6.08 and 7.80 µM for Fuyang573, SH12-036 and SH12-276, respectively (Figure 1B). Suramin was not cytotoxic at concentrations as high as 1 mM (Figure 1A) and had a selectivity index greater than 12 500. In TCID_50_ reduction assays, coxsackievirus A16 replication was reduced by 10^6^ TCID_50_/mL by 50 µM suramin, whereas poliovirus-1 (Sabin) was not inhibited (Supplementary Figure S2).

### Suramin inhibits EV71 entry

**Figure 2:**
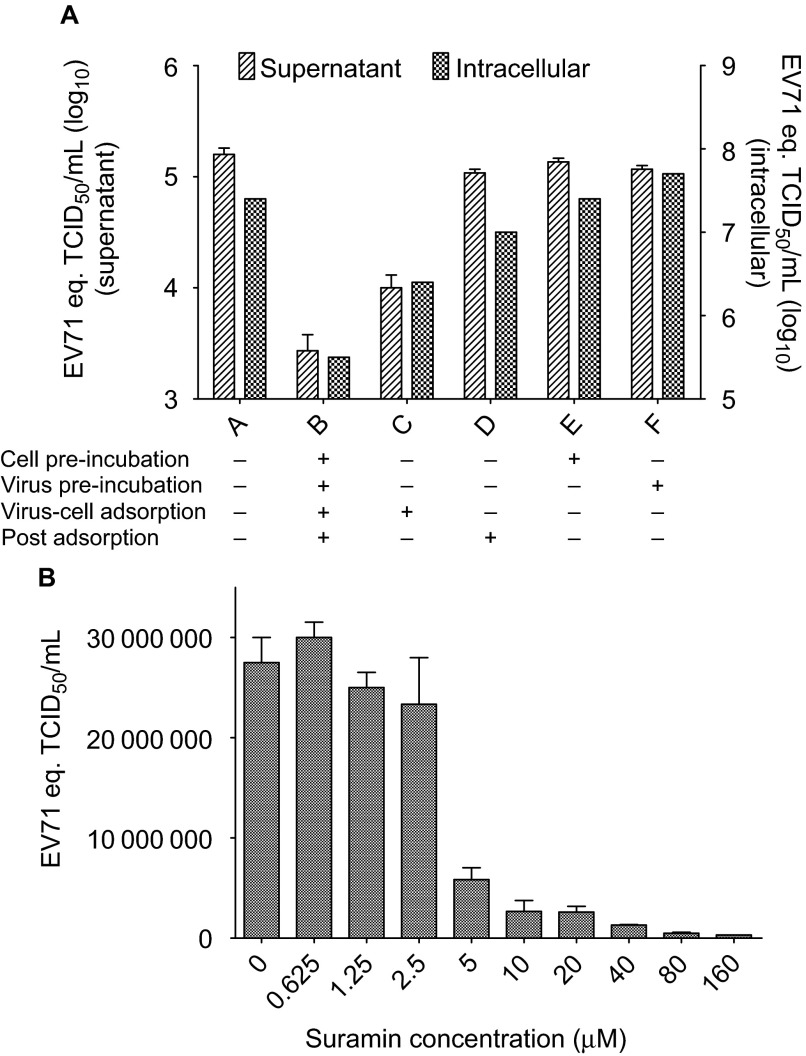
Suramin blocks EV71 virus-cell attachment. (**A**) Time of addition assay. 10 µM suramin was added at different stage in viral infection as shown in figure. The viral load in supernatant represents the means±s.e.m. of results of experiment with three replicates, and the intracellular viral load represents the result of a single test. (**B**) Inhibition of viral attachment by suramin (cell-virus adsorption at 4°C). Data represents the means±s.e.m. of results of experiment with three replications.

We investigated the step in the viral infectious cycle targeted by suramin, by time-of-addition assays in which cells and virus were pre-incubated or not with 10 µM suramin, and 10 µM suramin was present or not in viral-cell adsorption and post adsorption stage of EV71 infection. Single round viral replication is get by infecting RD cell at multiplicity of infection of 5, collecting at 16 h post infection, and testing culture supernatant and intracellular RNA at 16 h post infection. Suramin decreased viral replication by >1 log_10_ when added at the virus-cell adsorption stage, but had no effect if added after adsorption (Figure 2A). Furthermore, when incubated with cells and virus at 4°C, to prevent virus internalization, suramin reduced virus binding to the cell with an IC_90_ of 6.17 µM (Figure 2B).

### Sulfonated and sulfated compounds inhibit EV71 infection

**Figure 3:**
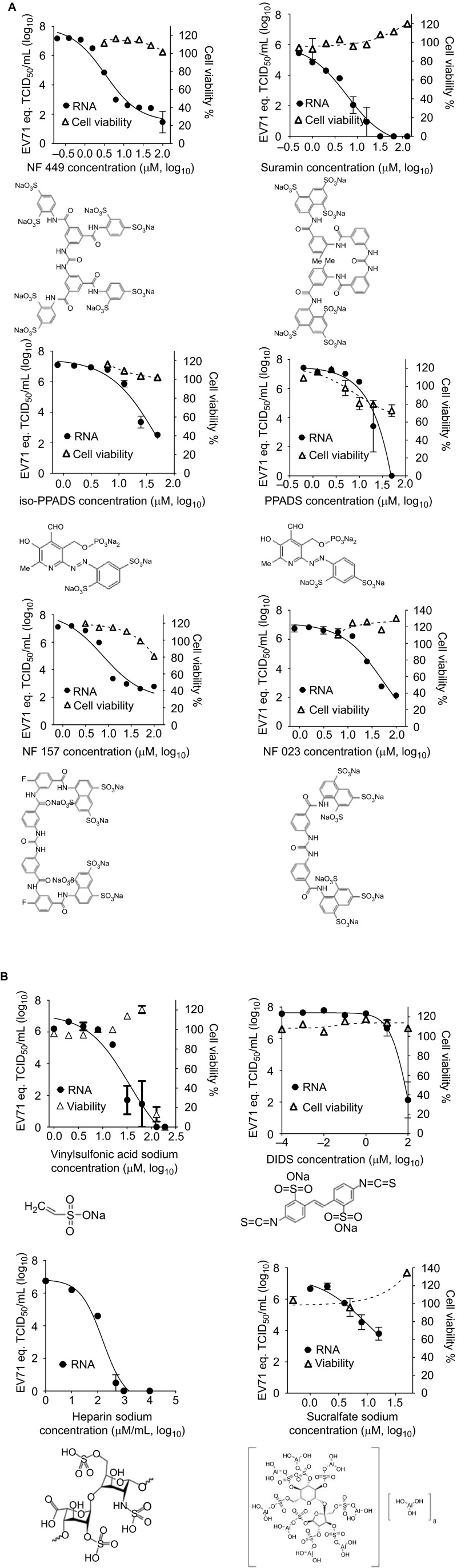
Sulfonated and sulfated compounds inhibit the replication of the EV71 Fuyang573 isolate. (**A**) Sulfated suramin analogs inhibit EV71 infection. Data represents the means±s.e.m. of results of experiment with three replications. (**B**) Sulfonated and sulfated compounds not analogous to suramin inhibit EV71 infection. Data represents the means±s.e.m. of results of experiment with three replications.

Structural analogs of suramin also inhibited EV71 replication (Figure 3A), with the following IC_90_: NF 449, 0.9 µM; NF 157, 2.5 µM; iso-PPADS, 6.4 µM; PPADS, 7.0 µM; NF 023, 8.9 µM. Sulfated and sulfonated compounds that were not structural analogs of suramin were also shown to be active: the monosulfonated compound vinylsulfonic acid sodium and the disulfonated compound DIDS inhibited EV71 infection with an IC_90_ of 4.5 µM and 10.3 µM, respectively, and the polysulfated molecules, heparin and sucralfate sodium, inhibited EV71 replication with IC_90_ values of 24.3 µg/mL and 3.3 µM, respectively.

**Figure 4:**
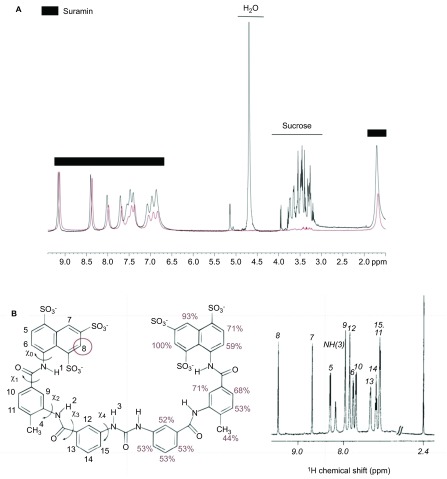
STD NMR assay of suramin-EV71 particle binding. (**A**) STD NMR of suramin with the EV71 particle. ^1^H (proton) NMR spectrum of suramin, shown in black, and STD NMR spectrum of suramin in complex with EV71 particles, shown in red. (**B**) Left: structure of suramin, labeled with proton positions and relative intensity percentages. Right: ^1^H NMR spectrum of suramin, with proton numbers shown.

STD NMR is a powerful tool for assessing small-molecule binding to viruses.^33^ We observed strong STD NMR signals for all protons on the suramin framework (Figure 4A), whereas the proton signals of added sucrose, an internal control, were not observed as anticipated in the STD NMR spectrum in the presence of inactivated, purified EV71. The suramin napthalenetrisulfonic acid moiety H7 and H8 protons, displayed the strongest signal intensities, indicating close proximity of these protons and consequently that part of the molecule to the EV71 capsid (Figure 4B).

Our results indicate that suramin binds to the EV71 particle via the naphthalenetrisulfonic acid group, preventing viral attachment and entry.

### Pharmacokinetics of suramin

CYP1A2 was the only CYP enzyme tested to display slight inhibition by suramin, with an IC_50_>10 µM (Supplementary Table S1), suggesting a low risk of drug–drug interaction. Suramin did not inhibit the human Ether-à-go-go-related gene channel (Supplementary Table S2), suggesting a low likelihood of cardiotoxicity.

The approved dose of suramin is 1 g for adults and 10–15 mg/kg for children (http://home.intekom.com/pharm/bayer/suramin.html). We used 15 mg/kg as the highest human reference dose. Following a single intravenous administration of 4.37 mg/kg suramin, corresponding to one-eleventh the highest human dose allometrically scaled to the monkey (46.5 mg/kg), in male cynomolgus monkeys, suramin was rapidly detected in the plasma and cleared slowly with an average CL of 0.0317 mL/min/kg (Supplementary Figure S3 and Supplementary Table S3). Suramin plasma level reached 10.9 µM at 24 h (Supplementary Figure S3 and Supplementary Table S4), which is >10 times superior to the *in vitro* IC_90_ (0.93 µM to Fuyang573 isolate) (Figure 1). Plasma drug level is 2.9 times to the IC_90_ at seven days (168 h) after a single-dose administration.

### Suramin efficacy

**Figure 5:**
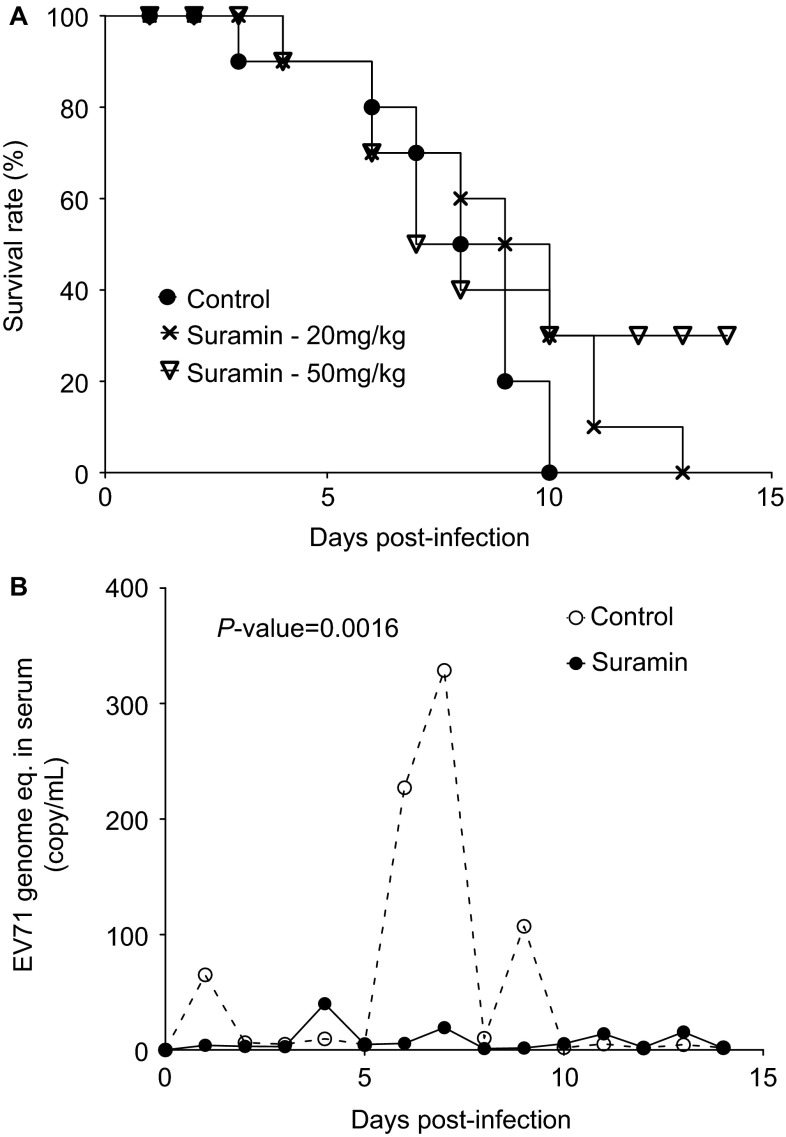
Anti-EV71 efficacy *in vivo*. (**A**) Infect 10-day-old ICR mice with 1×10^7^ TCID_50_ (lethal dose) of the mouse-adapted EV71 strain MP10. Then inject 20 or 50 mg/kg body weight suramin by i.p. injection, twice daily for 7 days. There are 10 mice in each group. (**B**) Suramin at a dose of 50 mg/kg body weight decreases EV71 viremia in adult rhesus monkeys. Challenge adult monkeys with 1×10^6.5^ CCID_50_ EV71 FY-23 strain, and inject 50 mg/kg suramin i.v., on the day before virus challenge and on days 1, 3 and 5 post challenge. There are five monkeys in each group. ICR, Institute of Cancer Research; i.p., intraperitoneal; i.v., intravenously.

We assessed the suramin efficacy in 10-day-old Institute of Cancer Research mice infected with lethal doses of the mouse-adapted EV71 strain MP10. Treatment with 50 mg/kg resulted in survival rates of 30% while vehicle-treated mice developed paralysis at 3 dpi and died within 10 days of infection (Figure 5A).

Rhesus and cynomolgus monkeys can be successfully infected with EV71 and represent the most predictive animal models for EV71.^30,34,35^ Rhesus monkeys were treated four times with 50 mg/kg suramin, the highest human dose allometrically scaled to the monkey (http://www.fda.gov/downloads/Drugs/GuidanceComplianceRegulatoryInformation/Guidances/ucm078932.pdf), at two-day intervals, starting one day before infection with 1×10^6.5^ CCID_50_ EV71 FY-23. In the vehicle-treated control group, more than 100 copies of EV71 genomic RNA per mL of blood were readily detected in sera at days 6 and 9 post-infection by qRT-PCR, with viremia peaking at 7 dpi. However, in the suramin-treated group, fewer than 20 copies of viral genomic RNA/mL were detected between days 6 and 9 (Figure 5B). EV71 neutralizing antibody was negative in all serum collected before viral challenge, monkeys involved in this study do not have pre-antibody. And all serum collected at 2, 3 and 3 weeks post challenge have EV71 neutralizing antibody.

These data demonstrate that suramin has a favorable pharmacokinetic and toxicity profile and inhibits EV71 replication *in vivo* at doses at or below the highest human dose.

## DISCUSSION

Drug development for acute pediatric infectious diseases is challenging due to long development times and high costs.^36^ We reasoned that the repurposing of approved drugs,^37^ particularly those for pediatric use, might be a useful approach. We found that suramin, previously approved for the treatment and prophylaxis of African sleeping sickness and onchocerciasis,^38^ inhibited EV71 replication *in vivo* at doses at or below the highest human dose.

HFMD epidemics occur annually in several Asian countries.^1,2^ Physicians are faced with large numbers of patients with mild symptoms (rash, fever)^8^ and a lack of markers of progression to severe disease, which typically occurs one to two days after symptom onset. There are two major therapeutic needs: (i) treatment of children diagnosed with EV71, to prevent progression to severe forms and death; and (ii) prophylactic treatment of children in contact with EV71-infected children, to prevent viral transmission.

Suramin was the only drug, approved for prophylactic and therapeutic uses in children, identified in our screening campaign. Significant toxicity has been observed in patients with Trypanosoma infection, due to inflammatory reactions caused by suramin-mediated killing of parasite.^39^ However, data from patients without parasitic infections^40,41,42^ suggest that suramin is generally safe, provided that plasma drug concentrations do not exceed 200 μM.^40^ Furthermore, suramin had no significant effect on Ether-à-go-go-related gene channel activity and little potential for drug–drug interactions.

Suramin has a long half-life^25,43^ and >10 times the IC_90_ for EV71 can be reached in monkeys after a single injection at one-eleventh the highest human dose allometrically scaled to monkeys according to United States Food and Drug Administration guidelines. This profile makes a single-dose strategy possible, with sufficiently high drug concentrations being reached over a few days following a single injection and ensuring antiviral efficacy throughout the period of peak viremia.^30,35,44^

Most antiviral drugs target viral enzymes involved in replication, but viral entry has been successfully used as a target for antiviral drug development for human immunodeficiency virus.^45,46,47^ Pleconaril, which binds the capsid of human rhinovirus, a picornavirus, prevents virus entry.^48^ It was tested in phase III trials for common cold treatment, but did not obtain regulatory approval. Pleconaril is also active against EV71 but its potency varies considerably between viral isolates.

The mode of action of suramin involves the sulfonate groups of the naphthalene moiety. Our results are consistent with those of Tan *et al*.,^14,22^ who simultaneously described sulfate-mediated inhibition of EV71 entry by demonstrating the binding of EV71 to cell surface heparan sulfate glycosaminoglycan and the blocking of this binding by suramin. NF449, a suramin analog, has also been shown to inhibit EV71.^49^ A large number of sulfated and sulfonated molecules inhibit EV71 (Figure 3), including several antagonists of P2X receptors (Figure 3A), suggesting a possible role for P2X receptors in cell entry. However, no non-sulfonated/sulfated P2X inhibitors displayed activity (Figure 3B) and the siRNA knockdown of P2X receptors did not decrease viral replication or affect the ability of suramin to block EV71 replication (data not shown).

Time-of-addition and virus binding assays showed that suramin prevented EV71 from binding to the target cell *in vitro* (Figure 2). STD NMR spectroscopy is a powerful tool for identifying the pharmacophores of small molecules binding to virus particles.^33^ Our study of suramin in complex with EV71 particles by this technique clearly demonstrated that the protons adjacent to the viral capsid are positioned close to the sulfonic acid groups, identifying the naphthalene trisulfonic acid group as the pharmacophore by which suramin binds to and inhibits virus attachment and replication (Figure 4). Mechanism-of-action studies suggested that suramin inhibited virus entry through a mechanism similar to the antibody-mediated neutralization of virus particles.

We evaluated suramin efficacy in two validated animal models.^29,30^ In mice,^29^ suramin decreased mortality by 30% (Figure 5A). In the monkey model, previously shown to be of predictive value in vaccine development,^31^ the highest human dose of suramin, allometrically scaled to the monkey decreased peak viremia (Figure 5B). This provides the first proof-of-concept that a small-molecule inhibitor can have a strong antiviral effect against EV71 in non-human primates.

Suramin displays high levels of serum protein binding, generally considered predictive of poor therapeutic efficacy for small molecules.^50^ However, our data suggest that the protein-binding features of suramin may be a key element in its anti-EV71 activity and that circulating EV71 may be neutralized by suramin the blood.

The primary objective of the treatment of EV71 infection is preventing severe and fatal outcome of disease. Our findings suggest that suramin, an approved pediatric drug, may be useful for therapeutic and prophylactic applications in young children infected with or exposed to EV71. Overall, this study indicates that the identification of new indications for approved drugs is an attractive approach for developing new treatments for acute viral infections in situations of major unmet need. Moreover, we believe that our study supports the notion that suramin presents an exciting opportunity as a possible drug candidate to treat and prevent HFMD and severe EV71 infections. This opportunity should be investigated further, by evaluating safety and efficacy in clinical studies.
