# Supplementary material for: The approved pediatric drug suramin identified as a clinical candidate for the treatment of EV71 infection—suramin inhibits EV71 infection in vitro and in vivo
Source: Emerg Microbes Infect. 2014 Sep 3;3(9):e62–. doi: 10.1038/emi.2014.60 (PMC4185360; doi:10.1038/emi.2014.60)
Supplement: Supplementary Table S1 [file emi201460x1.pdf]

Supplementary Table S1 CYP inhibition assay for suramin

	1A2		2C9		2C19		2D6		3A4	
**Conc (μM)**	Inhibition	IC_{50} (μM)	Inhibition	IC_{50} (μM)	Inhibition	IC_{50} (μM)	Inhibition	IC_{50} (μM)	Inhibition	IC_{50} (μM)
100	89%	12.3	32%	>100	56%	78.9	49%	>100	34%	>100
30	70%	12.7	8%	>100	34%	58.9	40%	45.5	16%	>100
10	40%	15.1	0%	//	15%	56.9	7%	>100	5%	>100
