# Supplementary material for: The approved pediatric drug suramin identified as a clinical candidate for the treatment of EV71 infection—suramin inhibits EV71 infection in vitro and in vivo
Source: Emerg Microbes Infect. 2014 Sep 3;3(9):e62–. doi: 10.1038/emi.2014.60 (PMC4185360; doi:10.1038/emi.2014.60)
Supplement: Supplementary Table S2 [file emi201460x2.pdf]

Supplementary Table S2 hERG channel inhibition by suramin

Compound	IC_{50}_hERG (μM)
**Amitriptyline**	4.04
**Suramin**	>1000.00
