# Supplementary material for: The approved pediatric drug suramin identified as a clinical candidate for the treatment of EV71 infection—suramin inhibits EV71 infection in vitro and in vivo
Source: Emerg Microbes Infect. 2014 Sep 3;3(9):e62–. doi: 10.1038/emi.2014.60 (PMC4185360; doi:10.1038/emi.2014.60)
Supplement: Supplementary Table S3 [file emi201460x3.pdf]

Supplementary Table S3 Individual and mean pharmacokinetic parameters of surmanin following single intravenous bolus administration of suramin at 4.37mg/kg to male cynolmolgus monkeys

Animal ID	Cmax (ng/mL)	Tmax (h)	C0 (ng/mL)	T1/2 (h)	Vdss (L/kg)	Cl (mL/min/kg)	AUC0-last (ng·h/mL)	AUC0-inf (ng·h/mL)
P101	169000	0.08	143000	69.06	0.176	0.0371	1660000	1960000
P102	117000	0.08	96300	112.49	0.212	0.0303	1810000	2400000
P103	141000	0.03	146000	130.31	0.210	0.0278	1930000	2620000
Mean	142333	0.06	128433	103.95	0.199	0.0317	1800000	2326667
SD	26026	0.03	27869	31.50	0.0202	0.00481	135277	336056
CV (%)	18.3	45.6	21.7	30.3	10.1	15.2	7.52	14.4

The linear regression coefficient of the concentration values on the terminal phase is less than 0.9. t1/2 might not be accurately estimated.
