# Supplementary material for: The approved pediatric drug suramin identified as a clinical candidate for the treatment of EV71 infection—suramin inhibits EV71 infection in vitro and in vivo
Source: Emerg Microbes Infect. 2014 Sep 3;3(9):e62–. doi: 10.1038/emi.2014.60 (PMC4185360; doi:10.1038/emi.2014.60)
Supplement: Supplementary Table S4 [file emi201460x4.pdf]

Supplementary Table S4 Individual and mean plasma concentration of surmanin following single intravenous bolus administration of suramin at 4.37mg/kg to male cynolmolgus monkeys.

Time (h)	Conc. (μM)			
	**P101**	**P102**	**P103**	**Mean**
0	BQL	BQL	BQL	BQL
0.033	110.00	74.20	109.00	97.73
0.083	130.00	90.50	104.00	108.17
0.25	103.00	79.90	93.10	92.00
0.5	103.00	82.10	92.30	92.47
1	77.80	66.70	85.50	76.67
3	54.00	48.90	53.80	52.23
6	32.20	35.20	37.10	34.83
9	18.30	23.20	25.80	22.43
24	9.83	11.30	11.60	10.91
48	6.20	7.11	8.61	7.31
72	4.38	5.55	5.31	5.08
96	3.39	4.44	4.18	4.00
120	3.74	3.79	3.64	3.72
144	3.42	3.23	3.16	3.27
168	2.31	2.82	2.82	2.65

Note: BQL- Below the lower limit of quantitation (LLOQ).
