# Supplementary material for: The approved pediatric drug suramin identified as a clinical candidate for the treatment of EV71 infection—suramin inhibits EV71 infection in vitro and in vivo
Source: Emerg Microbes Infect. 2014 Sep 3;3(9):e62–. doi: 10.1038/emi.2014.60 (PMC4185360; doi:10.1038/emi.2014.60)
Supplement: Supplementary Figure S1 [file emi201460x5.pdf]

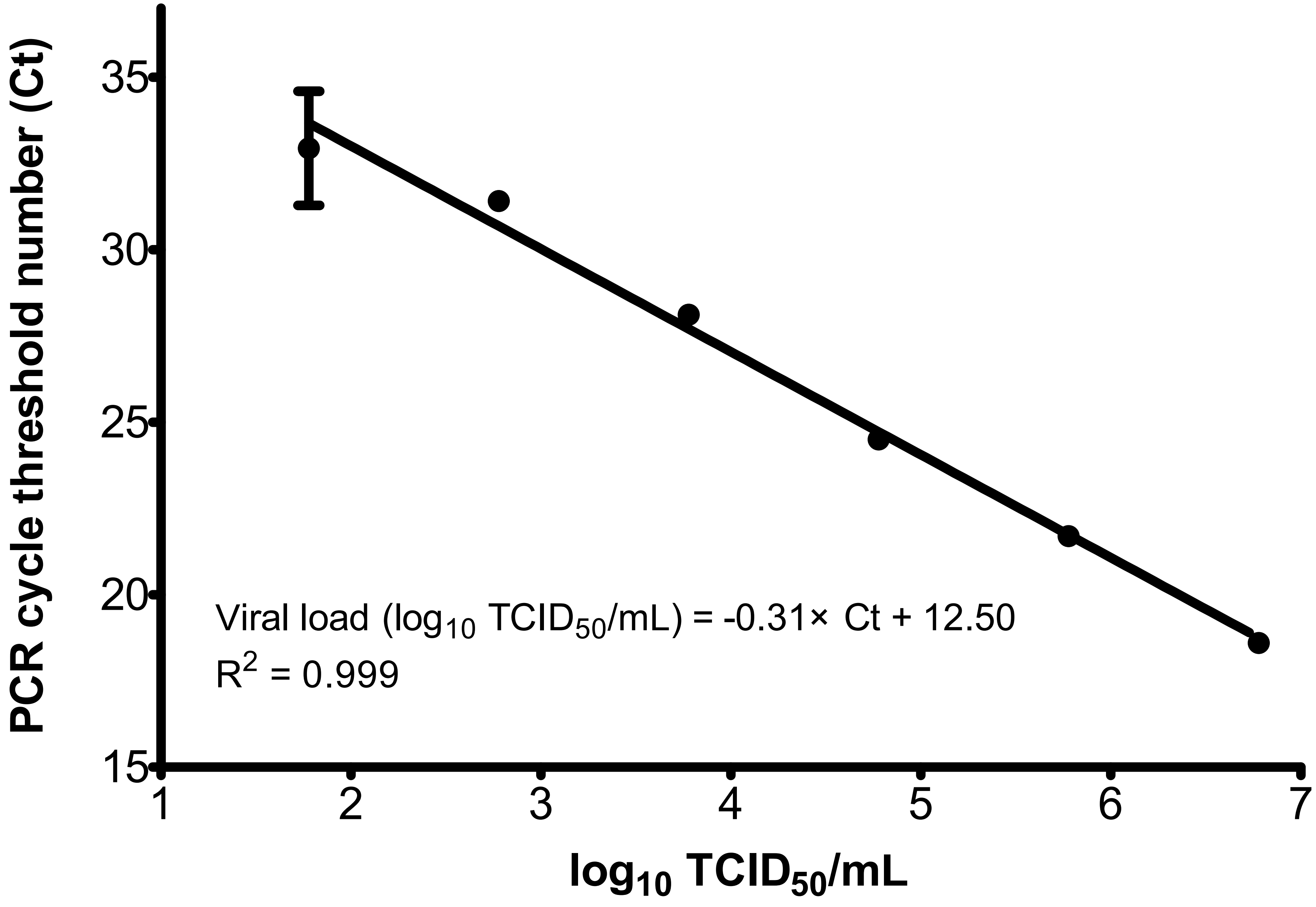

PCR cycle threshold number (Ct)
35
30
25
20
15
1
2
3
4
5
6
7
Viral load (log_{10} $TCID_{50}$/mL) = -0.31× Ct + 12.50
R^2 = 0.999
log_{10} $TCID_{50}$/mL

Supplementary Figure S1 Correlation between EV71 genome PCR CT and viral load. PCR standard curve was run for each viral quantification test. Dilute virus solution with defined titer ($TCID_{50}$/mL) serially by 10 times, extract viral RNA and apply to real time RT-PCR spontaneously with testing samples. Correlate the PCR CT with viral load to get the standard curve equation. Substitute PCR CT of testing sample into standard curve equation, so that the viral load in testing sample can be converted from PCR CT to viral load expressed by genome equivalence (eq.) to $TCID_{50}$/mL. Data represents the means ± SEM of results of duplicated experiment.
