# Supplementary material for: The approved pediatric drug suramin identified as a clinical candidate for the treatment of EV71 infection—suramin inhibits EV71 infection in vitro and in vivo
Source: Emerg Microbes Infect. 2014 Sep 3;3(9):e62–. doi: 10.1038/emi.2014.60 (PMC4185360; doi:10.1038/emi.2014.60)
Supplement: Supplementary Figure S2 [file emi201460x6.pdf]

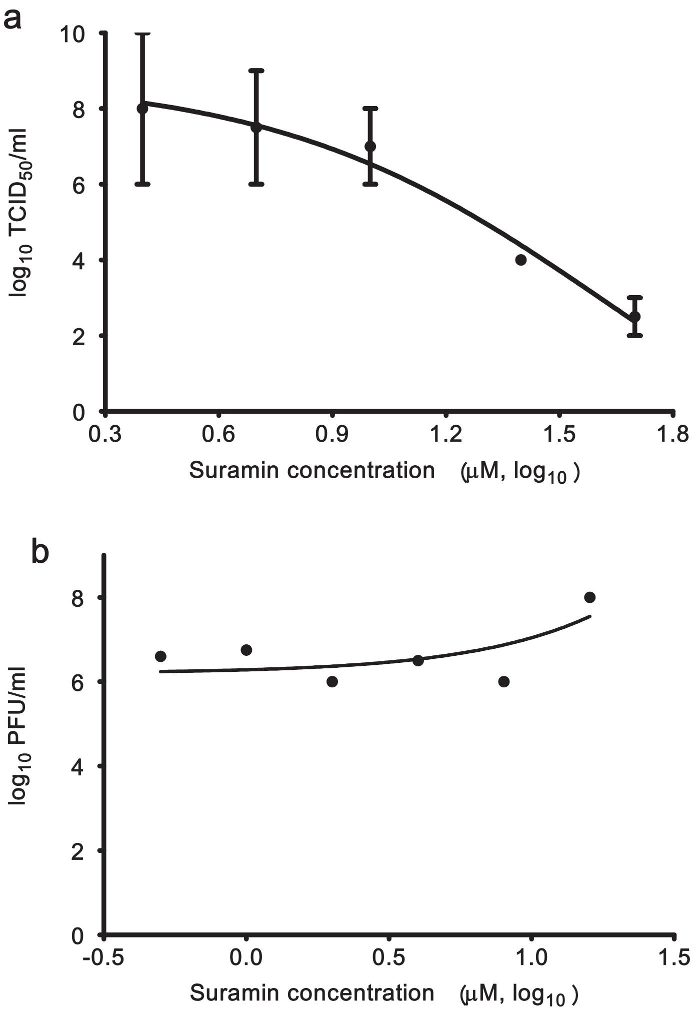

Supplementary Figure S2 Antiviral profile of suramin. (a) Suramin reduces CVA16 infectivity. Test was replicated, and the data represents the means +/- SEM of results. (b) Poliovirus-1 is not inhibited by suramin. Data represents the result of single test.
