# Supplementary material for: The approved pediatric drug suramin identified as a clinical candidate for the treatment of EV71 infection—suramin inhibits EV71 infection in vitro and in vivo
Source: Emerg Microbes Infect. 2014 Sep 3;3(9):e62–. doi: 10.1038/emi.2014.60 (PMC4185360; doi:10.1038/emi.2014.60)
Supplement: Supplementary Figure S3 [file emi201460x7.pdf]

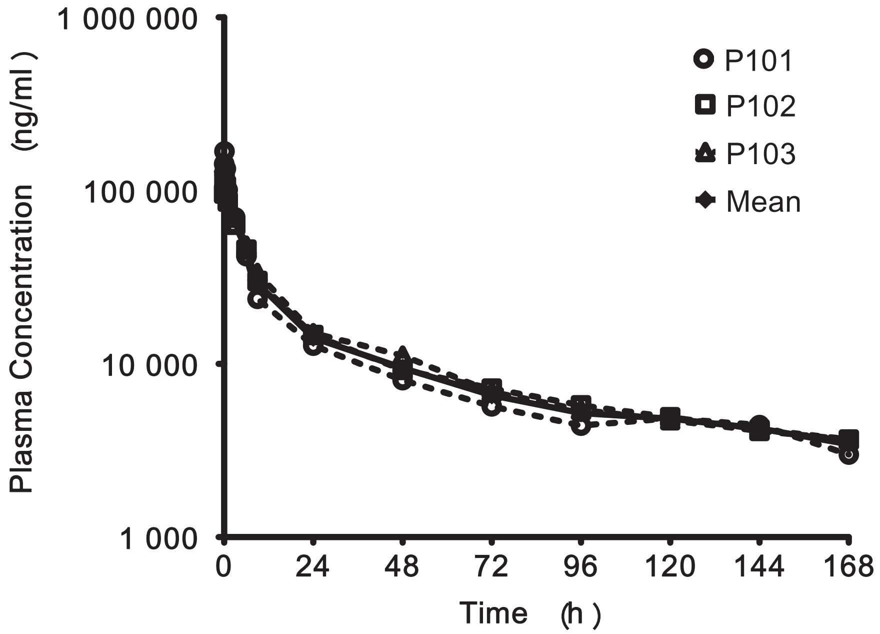

Supplementary Figure S3 Individual and mean plasma concentrations versus time profile of suramin following intravenous bolus administration of suramin at 4.37 mg/kg to male cynomolgus monkeys. Three monkeys were included, and were labeled as P101, P102 and P103 respectively.
